# Resistance strategies and attitude certainty in persuasion: bolstering vs. counterarguing

**DOI:** 10.3389/fpsyg.2023.1191293

**Published:** 2023-08-02

**Authors:** Kevin L. Blankenship, Marielle G. Machacek, Julia L. Standefer

**Affiliations:** Department of Psychology, Iowa State University, Ames, IA, United States

**Keywords:** resistance strategies, counterarguing, bolstering, attitude certainty, persuasion

## Abstract

**Introduction:**

Although resistance to persuasion has been of interest in psychology, relatively little research has examined how different resistance strategies can affect the strength-related features of attitudes. The current research presents a metacognitive account of two resistance strategies and their effect on attitude certainty and intentions. Specifically, we examine how the strategies of counterarguing and bolstering can differentially affect attitude certainty and intentions to act on the attitude under attack.

**Methods:**

In two experiments, we implemented a 2(Perceived Thought Type: bolster vs. counterargue) x 2(Perceived Argument Quality: weak vs. strong) between-participants design. Participants read weak or strong arguments about a counterattitudinal topic. After reporting their thoughts in response to the message topic, participants received bogus feedback regarding the nature of their thoughts (i.e., bolstering or counterarguing). Following the feedback, participants reported their attitudes and attitude certainty.

**Results:**

In Experiment 1 (*N* =241), participants’ thoughts perceived as counterarguments elicited attitude certainty that was more sensitive to the quality of the attacking information than when thoughts were perceived as bolstering one’s opinion. Experiment 2 (*N*  = 287) replicated the effect with a different topic and demonstrated a similar pattern on intentions to act on the attacked attitude.

**Discussion:**

The research demonstrates that two relatively thoughtful strategies, bolstering and counterarguing, can play an important role in attitude certainty and intentions following a persuasion attempt.

## Introduction

Relative to our understanding of the various conditions and mechanisms responsible for successful attitude change via persuasion (Petty and Cacioppo, 1996; Albarracín and Johnson, 2019; Nolder and Blankenship, 2019), less is known about resistance to persuasion. In some cases, resistance and persuasion have been treated as parallel constructs, with increases in one leading to decreases in the other (i.e., resistance as an outcome; Wegener et al., 2004). However, resistance *per se* is more complex than just the antithesis to attitude change. For example, resistance has been examined as a process (Killeya and Johnson, 1998), a motivation (Festinger, 1957; Brehm, 1966), a quality of a person (Rokeach, 1960; Saucier and Webster, 2010), an attitude (McGuire, 1964), and as a strategy (Jacks and Cameron, 2003). The present experiments examine an aspect of the last conceptualization, as they compare two commonly used resistance strategies and their influence on attitude certainty and intentions to act when one is confronted with counterattitudinal information.

### Resistance strategies in persuasion

While a number of strategies for resisting an appeal have been identified (Jacks and Cameron, 2003; Knowles and Linn, 2004; Fransen et al., 2015), our current focus is on those where message recipients generate issue-relevant thoughts in response to counterattitudinal information. Specifically, we examine how self-generated thoughts and their perceived relation to the attacking information can affect attitude certainty (Tormala and Petty, 2002). For example, consider someone who is against hydraulic fracking. When confronted with information that favors fracking, the individual may generate thoughts that support their initial opinion (e.g., “*fracking can contaminate drinking water*”). This type of thought (i.e., a bolstering thought; Lewan and Stotland, 1961; McGuire, 1964) serves to empower the attacked opinion and can create resistance by reinforcing one’s own stance on an issue. On the other hand, one may generate thoughts that directly refute the information that serves as the basis for the opposing position (McGuire, 1964; Brock, 1967; Greenwald, 1968). For example, a counterargument regarding the benefits of moving from coal-generated to natural gas-generated energy on air quality might be “*Air quality dynamics around fracking are not fully understood and natural gas is not a purely clean and renewable source of energy, and so its benefits are only relative*.” Counterarguments can create resistance by enhancing attitude strength features (e.g., certainty, Tormala and Petty, 2002). Importantly, compared to other strategies such as source derogation, bolstering and counterarguing strategies are used more frequently (Jacks and Cameron, 2003), and the amount of effort involved in implementing them is greater (i.e., effortful resistance strategies; Briñol et al., 2004; Wegener et al., 2004). Thus, use of these strategies may create a successful form of resistance that yields a durable and impactful attitude.

However, bolstering and counterarguing may differ in meaningful ways. One notable difference pertains to the extent to which an attack serves as a reference point to the thoughts generated in the persuasion context and any attributions made about the thoughts. Counterarguing requires refutation of information that challenges one’s opinion (i.e., a contesting strategy, Fransen et al., 2015). During this process, message recipients “size up” the attacking information and attempt to refute it (Brock, 1967). Thus, the attacking information can serve as a reference point for which there is an indirect comparison between the attacking information and the recipient’s refutations. From this comparison is derived the perceived success of defending the attack from refuting the information (Rucker and Petty, 2004), which can influence perceptions of attitude certainty (i.e., the subjective sense that one’s attitude is valid; Gross et al., 1995). Indeed, consistent with a metacognitive account of resistance (Tormala and Petty, 2004), perceived success of counterarguing depends on the strength of the attack (Tormala and Petty, 2002). In a series of experiments, Tormala and Petty (2002) instructed participants to counterargue a counterattitudinal message that varied in argument quality. Participants were told that the message contained weak and specious reasons or compelling reasons (Exp. 1), or actually contained weak and specious reasons or compelling reasons (Exp. 2). Although post-message attitudes were similar across the argument quality conditions, counterarguing affected attitude certainty as a function of argument quality, such that counterarguing a compelling message (perceived or actual) resulted in greater attitude certainty than counterarguing a weak message. Thus, successfully refuting a strong attack, compared to a weak one, is a better indicator of one’s attitude conviction, resulting in subsequent appraisals of that attitude being “battle-tested” and diagnostic of one’s ability to fend off future attacks.

On the other hand, bolstering is a resistance strategy where the generation of issue-relevant thoughts are more reliant on the quality of the supportive thoughts than of the attacking information (i.e., an empowerment strategy; Fransen et al., 2015). Bolstering thoughts that are representative, most accessible, and strongest are those that come to mind (Janis and King, 1954). That is, while the cogency of bolstering thoughts is likely to be similar to those framed as counterarguments (Greenwald and Albert, 1968), bolstering thoughts are less reliant on assessing the strength of the attack (e.g., argument quality). As a result, the perceived effectiveness of resisting through bolstering (e.g., attitude certainty) may not be as sensitive to the quality of the attacking information as counterarguments because the thoughts are focused on the issue and one’s opinion more than the attacking information. In applying this reasoning to the current work, the perception that one has generated bolstering thoughts may generate attitude certainty that is less affected by the quality of the attack, relative to the perception that one has generated counterarguments.

### The present research

We examine whether two commonly used effortful resistance strategies differ in attitude certainty they generate following a strong vs. weak attack. Specifically, we examine whether perceived resistance strategy type moderates the effect of an attack’s perceived argument quality on attitude certainty. We hypothesize that, consistent with Tormala and Petty (2002), when thoughts are perceived as counterarguments, exposure to strong arguments in a counterattitudinal message would yield greater attitude certainty than weak arguments. However, when thoughts are perceived as bolstering, there would be no difference in attitude certainty as a function of perceived argument quality. These experiments test these hypotheses from an appraisal-based framework (Tormala and Petty, 2004; Rucker et al., 2014), such that the resistance strategies made salient can affect an attitude-strength feature such as certainty, despite the attitude not differing as a function of argument quality. Specifically, we examine thought bolstering and counterarguing resistance strategies and their influence on attitude certainty and intentions to act on one’s attitude.

In two experiments, participants read about and reported their thoughts regarding a counterattitudinal topic. To test whether the resistance strategies influence attitude certainty, we implemented a bogus feedback paradigm that manipulated the type of thoughts generated by participants (i.e., bolstering vs. counterarguments). It is expected that, despite similar levels of effort in generating thoughts, thoughts perceived as bolstering would yield attitude certainty that is less affected by quality of the attacking information, relative to thoughts framed as counterarguments, where exposure to strong arguments would yield greater attitude certainty than exposure to weak arguments. Such a finding would be the first evidence to show the limiting effect of argument quality on certainty across the different types of thought-based resistance strategies one could potentially have.

To test these hypotheses, we based our sample size on previous research. For Experiment 1, we aimed for at least 50 participants per condition, which is consistent with previous research on attitude certainty and counterarguing (Tormala and Petty, 2002). For both experiments, we also used a time-based stopping rule by collecting data until the end of the semester, with the assumption that we would attain at least 50 participants per condition.^1^ With these sample sizes, an effect size of *f* = 0.17 can be detected with 80% power. Data and materials are available here: https://osf.io/jc5n3/?view_only=a772c2458e884137805491450f115a28.

## Experiment 1

In Experiment 1, we provide an initial test of the moderating role of perceived resistance strategy type on perceived argument quality and certainty. Specifically, we expect that participants’ attitude certainty would be more sensitive to the quality of an attacking message when participants are told that their post-message thoughts counterargue the message rather than bolster their initial attitude. When participants are told that their thoughts bolster their initial attitude, however, it is expected that participants will report similar levels of certainty across the argument quality conditions. To test this, participants who support drug testing for welfare recipients were exposed to a counterattitudinal message (i.e., an anti-drug testing message). In order to manipulate the perceived resistance strategy, we adapted a bogus feedback paradigm (Tormala and Petty, 2002), such that participants received feedback regarding whether their thoughts were of the bolstering or counterarguing type.

### Participants and design

The study sample came from a larger data collection effort, where 524 students were recruited from a social science pool at a large Midwestern university. Of these, 246 reported favorable opinions toward the target issue of drug testing for welfare participants. Five additional participants were excluded from analyses because they did not write any thoughts following exposure to the message. The remaining 241 (159 female, 82 male *M_age_* = 19.73, *SD_age_* = 1.81) constituted the study sample for the 2(Perceived Thought Type: bolster vs. counterargue) × 2(Perceived Argument Quality: weak vs. strong) between-participants design.

### Procedure

The study was administered online via Qualtrics where all manipulations and measures were implemented. Participants were told that the study’s purpose was to gather students’ reactions toward various topics and issues in order to construct an opinion profile of the student body. Consistent with the cover story, participants reported their opinions toward the target issue of drug testing for welfare recipients and two filler issues of universal healthcare and fracking on separate 2-point scales (oppose vs. favor). As previously mentioned, because we were interested in resistance to persuasion, only participants who reported favoring drug testing for welfare recipients (*N* = 246) were exposed to the research materials. After reporting their opinions, participants read a counterattitudinal message that opposed drug testing for welfare recipients. Just before reading the message, participants were exposed to the argument quality manipulation such that half of participants were told that the message contained strong arguments whereas half were told that the message contained weak arguments (Tormala and Petty, 2002). Thus, we manipulated the *perceived* quality of the persuasive message, such that participants were led to believe that the message was either strong or weak.

Directly following the manipulation, all participants read the same message opposing drug testing for welfare recipients that contained three arguments. The arguments are summarized as: (a) most welfare recipients spend the welfare money on necessities, (b) drug use is no more a problem for individuals receiving welfare than people who do not, and (c) such laws discriminate based on socioeconomic status. The arguments were presented on separate screens in that order. After reading all three arguments, participants were given an opportunity to write up to four thoughts about the information. Participants were then told that the computer would analyze their thoughts and the analysis would indicate (via bogus feedback) the nature of their thoughts (i.e., the bolstering type or the counterarguing type). Specifically, participants in the bolstering conditions were provided information indicating their thoughts supported their initial opinion, whereas participants in the counterarguing conditions were given information indicating their thoughts refuted the message content. Participants across both conditions received positive feedback such that their thoughts provided a great deal of support for their opinions (bolstering), or that their thoughts counterargued the message very well.

Following the feedback, participants reported their attitudes, attitude certainty, perceived engagement with the issue, and an argument quality manipulation check. Participants were then debriefed as to the fictitious nature of the message and the purpose of the research.

### Independent variables

#### Perceived thought type

After reporting their thoughts, participants received feedback regarding the nature of their thoughts (i.e., bolstering or counterarguing). In the bolstering conditions, participants read “*… the computer will measure the extent to which your thoughts support your initial position toward drug testing for welfare recipients.*” In the counterarguing conditions, participants read: “*… the computer will measure the extent to which your thoughts refute the claims in the message.*” Participants were told that the index could range from 1 to 10, with higher scores indicating greater bolster or counterarguing, depending on their assigned condition.

After an 8-s delay used to simulate the basis calculation, the computer provided participants with a number, labeled as either a “*thought bolstering index*” or “*counterarguing index*.” The number was provided along with corresponding information about the level of bolstering or counterarguing. All participants received a 9 (out of 10) and told that their thoughts provided a great deal of support for their initial opinion (bolster conditions) or counterargued the message very well (counterargue conditions).

#### Perceived argument quality

All participants received the same message. Prior to reading the message, however, participants were told that the study was interested in getting reactions to all types of reasons that oppose drug testing for welfare recipients. Participants in the weak argument conditions were told that they will read the weakest reasons opposing drug testing, whereas participants in the strong conditions were told that they will read the strongest reasons (see Tormala and Petty, 2002 for a similar manipulation).

### Dependent variables

#### Thoughts

After reading the message, participants were given up to four boxes and were instructed to type one thought per box. Importantly, the thought listing instructions simply asked participants to list their thoughts; the instructions did not guide participants to list either bolstering our counterarguing thoughts.

#### Attitudes

Following the feedback manipulation, participants reported their attitudes toward drug testing welfare recipients on five 9-point scales (1 = *do not approve, unfavorable, bad, harmful, foolish,* 9 = *very much approve, favorable, good, beneficial,* and *wise*, respectively, *α* = 0.96).

#### Attitude certainty

Participants completed the four attitude clarity (e.g., “*How certain are you that you know what your true attitude on this topic really is?*”) and three attitude correctness items (e.g., *“How certain are you that your attitude toward drug testing for welfare recipients is the correct attitude to have?*”) adapted by Petrocelli et al. (2007; see also Cheatham and Tormala, 2015) on 9-point scales (1 = *not certain at all*, 9 = *very certain*). Items were presented randomly; both clarity (*α* = 0.96) and correctness (α = 0.83) demonstrated adequate reliability. All seven items were combined to create a single index of attitude certainty (*α* = 0.93).

#### Issue engagement

Previous research has suggested that bolstering and counterarguing resistance strategies are similarly effortful (Eagly and Chaiken, 1995; Briñol et al., 2004). However, any difference in self-reported effort stemming from the feedback manipulation may confound and undermine any observed differences in attitude certainty. Therefore, in order to test for any potential differences in this study, we assessed issue engagement on four 9-point scales. Specifically, following the attitude and attitude certainty items, participants reported how much attention they paid to the message (1 = *no attention at all*; 9 = *a lot of attention*), how deeply they thought about the message (1 = *not deeply at all*; 9 = *very deeply*), how much effort they put into reading the message (1 = *no effort at all*; 9 = *a lot of effort*), and how personally involved they felt with the topic (1 = *not involved at all*; 9 = *very involved*). Responses were combined to create a single index of issue engagement (*α* = 0.83; see also Tormala et al., 2006).

#### Perceived argument quality

Participants were asked to report how persuasive the message arguments were (1 = *not very persuasive*; 9 = *very persuasive*). This served as a manipulation check.

## Results

Along with additional descriptive statistics described below and reported in Table 1, a series of 2(Perceived Thought Type: bolster vs. counterargue) × 2(Perceived Argument Quality: weak vs. strong) between-participant Analysis of Variance (ANOVA) tests were conducted to examine our hypotheses.

**Table 1:** Experiment 1—dependent measures as a function of perceived thought type and perceived argument quality.

Measures	Perceived thought type					
	Bolster			Counterargue		
	Perceived argument quality			Perceived argument quality		
	Weak (*n* = 63) *M* (*SD*)	Strong (*n* = 60) *M* (*SD*)	*d*	Weak (*n* = 58) *M* (*SD*)	Strong (*n* = 60) *M* (*SD*)	*d*
Thought fav.	−0.10 (0.51)	−0.04 (0.41)	0.12	−0.06 (0.4)	−0.17 (0.35)	0.28
Attitudes	7.03 (1.98)	6.8 (1.78)	0.12	6.2 (1.88)	6.62 (2.22)	0.2
Attitude certainty	6.66 (1.6)	6.53 (1.69)	0.08	6.12 (1.71)	7.0 (1.64)	0.53*
Issue engagement	6.23 (1.56)	6.74 (1.4)	0.35	6.41 (1.54)	6.83 (1.54)	0.27
Perceived arg. strength	4.38 (1.93)	5.07 (2.01)	0.35	4.53 (2.13)	5.03 (2.34)	0.23

*Difference between argument quality within thought type *p* < 0.05.

### Dependent variables

#### Argument quality manipulation check

A 2(Perceived Thought Type: bolster vs. counterargue) × 2(Perceived Argument Quality: weak vs. strong) ANOVA on the argument quality manipulation check revealed a main effect of Perceived Argument Quality *F*(1, 237) = 4.71, *p* = 0.03, *d* = 0.28, with participants in the strong argument conditions rating the arguments as more persuasive (*M* = 5.05, *SD* = 2.17) than the arguments in the weak argument conditions (*M* = 4.45, *SD* = 2.02). No other effects were significant (*p*s > 0.73).

#### Thought favorability

Overall, participants generated an average of 2.68 thoughts (*SD* = 1.02) after reading the proposal. A research assistant blind to the study’s purpose and experimental conditions rated whether each thought was in favor (coded as +1), against (coded as −1), or neutral/irrelevant (coded as 0) toward the message. Thought favorability was calculated by subtracting the number of thoughts against the proposal from the thoughts favoring the proposal, divided by the total number of thoughts generated (see Wegener et al., 1995). Negative numbers suggest greater rejection of the message position (i.e., negative thoughts indicative of favoring drug testing). The message elicited negative thoughts (*M* = −0.09, *SD* = 0.42).

A 2(Perceived Thought Type: bolster vs. counterargue) × 2(Perceived Argument Quality: weak vs. strong) ANOVA on the thought favorability measure revealed no significant effects (*p*s > 0.14), suggesting any effect on attitude certainty is unlikely to be due to differences in thought favorability across the conditions.

#### Attitudes

The same analysis on participants’ attitudes revealed a marginal effect of Perceived Thought Type, *F*(1, 237) = 3.93, *p* = 0.05, *d* = 0.26, such that participants in the bolstering feedback conditions had more favorable attitudes (*M* = 6.91, *SD* = 1.88) than the counterargument feedback conditions (*M* = 6.41, *SD* = 2.06). No other effects were significant (*p*s > 0.20).

#### Attitude certainty

Consistent with previous research on argument quality and attitude certainty, we expected that participants would be less certain in their attitude after reading the weak message (Tormala and Petty, 2002). Importantly, we expect that this will occur only for participants in the counterarguing conditions.

**Figure 1:**
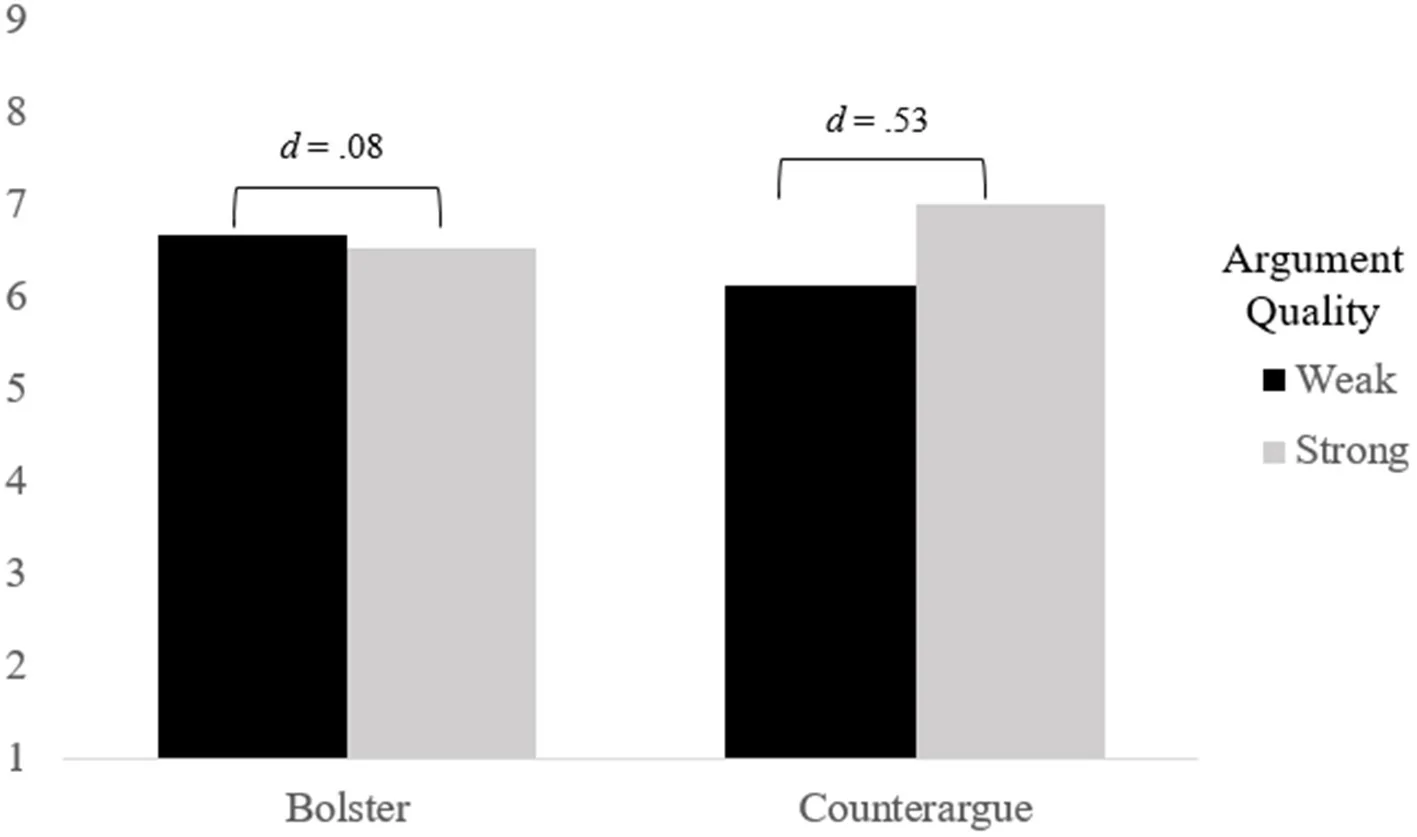
Experiment 1—attitude certainty as a function of perceived thought type and perceived argument quality.

To test this, we submitted the attitude certainty measure to a 2(Perceived Thought Type) × 2(Perceived Argument Quality) ANOVA. A marginal main effect of Perceived Argument Quality emerged *F*(1, 237) = 3.15, *p* = 0.08, *d* = 0.22, with participants reporting greater certainty following exposure to the message labeled as strong (*M* = 6.77, *SD* = 1.68) rather than weak (*M* = 6.4, *SD* = 1.67). Of greater importance was the significant Perceived Thought Type × Perceived Argument Quality interaction *F*(1, 237) = 5.71, *p* = 0.02, *n_p_*^2^ = 0.02 (see Figure 1). Specifically, in the bolstering conditions, there was no difference in attitude certainty when the message was labeled as weak (*M* = 6.66, *SD* = 1.60) or strong (*M* = 6.53, *SD* = 1.69), *F*(1, 237) = 0.02, *p* = 0.66, *d* = 0.08. However, in the counterarguing conditions, participants were more certain when the message was labeled as strong (*M* = 7.01, *SD* = 1.64) rather than weak (*M* = 6.12, *SD* = 1.71), *F*(1, 237) = 8.48, *p* = 0.004, *d* = 0.53. Thus, as predicted, perceived resistance strategy moderated the influence of argument quality on attitude certainty.

#### Issue engagement

Both bolstering and counterarguing have been speculated to be relatively high effort processes (Jacks and Cameron, 2003 Briñol et al., 2004). Indeed, inspection of the sample mean suggests that participants reported a relatively high level of message elaboration that was above the scale midpoint of 5.0 [*M* = 6.54, *SD* = 1.52; *t*(240) = 15.79, *p* < 0.001]. Nonetheless, we sought to rule out the possibility that any differences in attitude certainty may be due to differences in participants’ perceptions that they effortfully processed the message.

A 2(Perceived Thought Type) × 2(Perceived Argument Quality) ANOVA on the composite subjective elaboration measure revealed a main effect of Perceived Argument Quality *F*(1, 237) = 5.70, *p* = 0.02, *d* = 0.31, with participants reporting greater issue engagement following exposure to the message labeled as strong (*M* = 6.78, *SD* = 1.46) rather than weak (*M* = 6.31, *SD* = 1.55). No other effects were significant (*p*s > 0.48).

## Discussion

To recap, Experiment 1 demonstrated that attitude certainty resulting from thoughts perceived as counterarguments are affected by the quality of the attacking message, such that attitude certainty in response to a strong attack was higher than in response to a weak attack. However (and more novel), argument quality did not affect attitude certainty when thoughts were framed as bolstering thoughts. Moreover, similarly high levels of issue engagement were found across the thought framing conditions, suggesting that participants’ amount of processing was not responsible for these effects.

## Experiment 2

Having demonstrated the general finding, Experiment 2 built upon Experiment 1 in two ways. First, we sought to conceptually replicate Experiment 1 in a laboratory setting where experimental control is higher than in online-administered studies (Sargis et al., 2014). We also used a different counterattitudinal topic. Specifically, we used the issue of university service, as it has been found to be counterattitudinal for a student participant sample (e.g., Baker and Petty, 1994).^2^

We also examined the implications of thought type and argument quality on the relationship between attitudes and behavioral intentions. A common finding in the attitudes literature is that attitudes are more strongly related (i.e., impactful) to behavioral intentions to the extent that they are held with greater certainty (Fazio and Zanna, 1978; Gross et al., 1995; Barden and Petty, 2008). Thus, we predicted that attitudes and behavioral intentions would be more highly correlated for participants in the counterargument conditions, but only after participants resisted a persuasive attack believed to be strong. After resisting a persuasive attack believed to be weak, participants in the counterargument conditions would report less willingness to act on their attitude.

### Participants and design

Participants were 292 students recruited from a social science participant pool at a large Midwestern university. Five participants were excluded from analyses because they did not write any thoughts following exposure to the message. The remaining 287 (178 female, 109 male *M_age_* = 19.49, *SD_age_* = 1.7) constituted the study sample for the 2(Perceived Thought Type: bolster vs. counterargue) × 2(Perceived Argument Quality: weak vs. strong) between-participants design.

### Procedure

Participants were seated at a computer where the manipulation and measures were implemented. After consenting, all participants read the same message about a “university service” proposal that their university is considering implementing in the following academic year (see Baker and Petty, 1994, for a similar cover story and topic). If passed, the program would allow students to work for the university while enrolled. Students could opt out of the program but would have to pay out-of-state tuition. Before reading the message, participants were exposed to the argument quality manipulation from Experiment 1, such that half of participants were told that the message contained strong arguments whereas half were told that the message contained weak arguments.

Directly following the manipulation, all participants read four arguments supporting the proposal.^3^ After reading the proposal, participants were given an opportunity to write up to four thoughts. Similar to Experiment 1, participants were then told that the computer would analyze their thoughts and the analysis would indicate (via bogus feedback) the nature of their thoughts (i.e., the bolstering type or the counterarguing type).

Following the feedback, participants reported their attitudes, attitude certainty, perceived knowledge of the topic, perceived issue engagement, willingness to act, and various manipulation checks. Participants were then debriefed as to the fictitious nature of the message and the purpose of the research.

### Independent variables

#### Perceived thought type

We used the same feedback manipulation used in Experiment 1. That is, participants received feedback regarding the nature of their thoughts (i.e., bolstering thoughts or counterarguing thoughts).

After a 10-s delay used to simulate the basis calculation, the computer provided participants with a number, labeled as either a “*thought bolstering index*” or “*thought counterarguing index*.” The number was provided along with corresponding information about the level of bolstering or counterarguing. All participants received a 9 (out of 10) and told that their thoughts provided a great deal of support for their opinion (bolster conditions) or counterargued the message very well (counterargue conditions).

#### Perceived argument quality

Similar to Experiment 1, participants in the weak argument conditions were told that they will read the weakest reasons that support the program, whereas participants in the strong conditions were told that they will read the strongest reasons.

### Dependent variables

#### Thoughts

As with Experiment 1, participants were given up to four boxes and were instructed to type one thought per box. Importantly, the thought listing instructions simply asked participants to list their thoughts; the instructions did not guide participants to list either bolstering our counterarguing thoughts.

#### Attitudes

Following the bogus feedback manipulation, participants reported their attitudes toward the university service proposal using the same scales as in Experiment 1 (*α* = 0.96).

#### Attitude certainty

After reporting their attitudes, participants first reported their global attitude certainty on a 9-point scale (1 = *not at all*; 9 = *very much*). In addition, participants also completed the attitude clarity and attitude correctness items on the same 9-point scales from Experiment 1. Both clarity (*α* = 0.93) and correctness (*α* = 0.85) demonstrated adequate reliability. All eight items were combined to create a single index of attitude certainty (*α* = 0.93).

#### Perceived knowledge

Attitude-relevant knowledge refers to the extent to which one has information related to the attitude object of interest (Wood, 1982). Regardless of whether the knowledge is true or accurate, individuals with relatively high amounts of perceived knowledge tend to have attitudes that resist change (Wood et al., 1995). Previous correlational research found greater perceived knowledge to be associated with a counterarguing strategy (Jacks and Cameron, 2003; Study 3). For exploratory purposes, participants completed two items assessing their perceived knowledge about the program. Specifically, they were asked to report how much knowledge they have about the university service program and their knowledge about their attitude toward the university service program on 9-point scales (1 = *very little knowledge*; 9 = *a lot of knowledge*). The two items were combined to create an index of knowledge (*r* = 0.44*; p* < 0.001).

#### Issue engagement

Following the knowledge items, participants completed the subjective elaboration items using the same 9-point scales from Experiment 1 (*α* = 0.85).

#### Intentions

After reporting their issue engagement, participants reported their willingness to discuss their attitude toward the program (a) with someone who has an opposing viewpoint, (b) in public, and (c) their willingness to sign a petition that supports their attitude toward the university service program (1 = *not at all willing*; 9 = *very willing; α* = 0.80).

#### Perceived argument quality

Following the knowledge measures, participants were asked to report how persuasive the message arguments were (1 = *not very persuasive*; 9 = *very persuasive*).

## Results

Along with additional descriptive statistics described below and reported in Table 2, a series of 2(Perceived Thought Type: bolster vs. counterargue) × 2(Perceived Argument Quality: weak vs. strong) between-participant ANOVA tests were conducted to examine our hypotheses.

**Table 2:** Experiment 2—dependent measures as a function of perceived thought type and perceived argument quality.

Measures	Perceived thought type					
	Bolster			Counterargue		
	Perceived argument quality			Perceived argument quality		
	Weak (*n* = 72) *M* (*SD*)	Strong (*n* = 70) *M* (*SD*)	*d*	Weak (*n* = 72) *M* (*SD*)	Strong (*n* = 73) *M* (*SD*)	*d*
Attitudes	3.98 (2.37)	4.03 (2.07)	0.02	3.79 (1.76)	4.17 (1.86)	0.21
Thought fav.	−0.47 (0.7)	−0.49 (0.68)	0.03	−0.56 (0.58)	−0.53 (0.65)	0.06
Attitude certainty	6.62 (1.77)	6.58 (1.74)	0.02	6.38 (1.74)	6.93 (1.51)	0.53*
Perceived knowledge	5.14 (1.8)	5.19 (1.5)	0.03	4.64 (1.92)	5.6 (1.36)	0.58*
Issue engagement	7.01 (1.5)	7.15 (1.51)	0.09	7.03 (1.39)	7.42 (1.24)	0.29
Intentions	6.02 (2.02)	5.67 (2.2)	0.17	5.78 (1.9)	6.61 (1.83)	0.44*
Perceived arg. strength	3.66 (2.34)	4.47 (2.35)	0.35*	4.06 (2.14)	4.74 (2.3)	0.31

*Difference between argument quality within thought type *p* < 0.05.

### Dependent variables

#### Perceived argument quality

A 2(Perceived Thought Type: bolster vs. counterargue) × 2(Perceived Argument Quality: weak vs. strong) ANOVA on the argument quality manipulation check revealed a main effect of Perceived Argument Quality *F*(1, 283) = 7.79, *p* = 0.006, *d* = 0.33, with participants in the strong argument conditions rating the arguments as more persuasive (*M* = 4.61, *SD* = 2.31) than the arguments in the weak argument conditions (*M* = 3.85, *SD* = 2.24). No other effects were significant (*p*s > 0.21).

#### Thought favorability

Overall, participants generated an average of 3.08 thoughts (*SD* = 0.94) after reading the proposal. Following all of the critical dependent measures, participants rated each of their own thoughts in terms of its favorability toward the message. Specifically, participants rated whether each thought was in favor (coded as +1), against (coded as −1), or neutral/irrelevant (coded as 0) toward the proposal. Thought favorability was calculated by subtracting the number of thoughts against the proposal from the thoughts favoring the proposal, divided by the total number of thoughts generated (Wegener et al., 1995). Positive numbers suggest greater favorability toward the proposal. Consistent with previous research using a similar topic (Baker and Petty, 1994), the message elicited negative thoughts toward the proposal (*M* = −0.51, *SD* = 0.65).

A 2(Perceived Thought Type: bolster vs. counterargue) × 2(Perceived Argument Quality: weak vs. strong) ANOVA on the thought favorability measure revealed no significant effects (*p*s > 0.44), suggesting any effect on attitude certainty is unlikely to be due to differences in thought favorability across the conditions.

#### Attitudes

The same analysis on participants’ attitudes revealed no significant effects (*p*s > 0.36) suggesting that attitudes were not affected by the manipulations. Thus, any differences in attitude certainty are not likely due to changes in attitude favorability.

#### Attitude certainty

**Figure 2:**
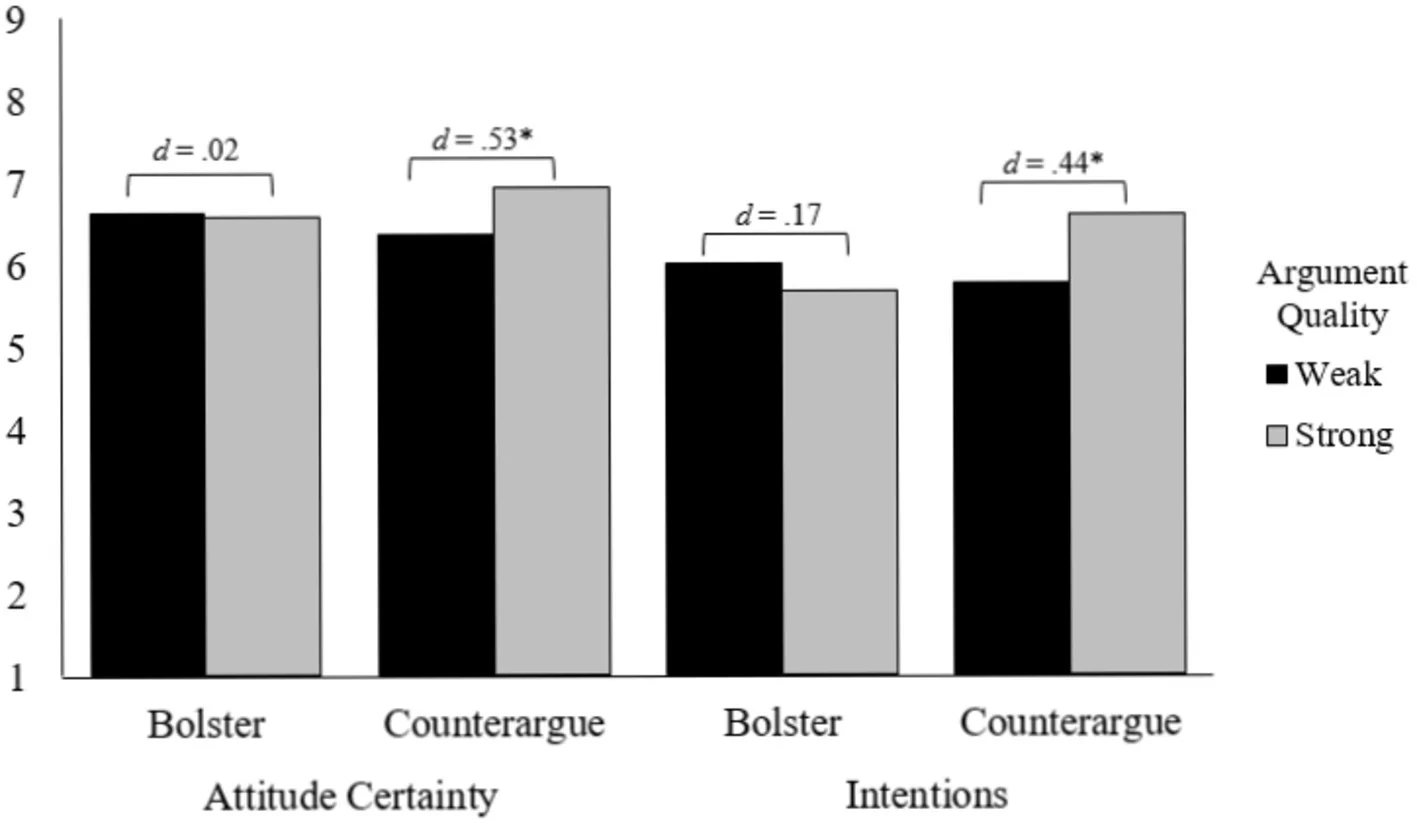
Experiment 2—attitude certainty as a function of perceived thought type and perceived argument quality. *p < .05.

We submitted the combined attitude certainty measure to a 2(Perceived Thought Type) × 2(Perceived Argument Quality) ANOVA. A marginal main effect of Perceived Argument Quality emerged *F*(1, 283) = 3.84, *p* = 0.05, *d* = 0.23, with participants reporting greater certainty following exposure to the message labeled as strong (*M* = 6.76, *SD* = 1.51) rather than weak (*M* = 6.38, *SD* = 1.74). Of greater importance was the significant Perceived Thought Type × Perceived Argument Quality interaction *F*(1, 283) = 4.61, *p* = 0.03, *n_p_*^2^ = 0.02 (see Figure 2). Specifically, in the bolstering conditions, there was no difference in attitude certainty when the message was labeled as weak (*M* = 6.62, *SD* = 1.77) or strong (*M* = 6.58, *SD* = 1.74), *F*(1, 283) = 0.02, *p* = 0.91, *d* = 0.02. However, in the counterarguing conditions, participants were more certain when the message was labeled as strong (*M* = 6.93, *SD* = 1.51) rather than weak (*M* = 6.38, *SD* = 1.74), *F*(1, 283) = 8.52, *p* = 0.004, *d* = 0.53.^4^ Thus, as predicted, perceived resistance strategy moderated the influence of argument quality on attitude certainty.

#### Knowledge

A 2(Perceived Thought Type) × 2(Perceived Argument Quality) ANOVA on the knowledge measure revealed a main effect of Perceived Argument Quality *F*(1, 283) = 6.70, *p* = 0.01, *d* = 0.30, with participants reporting greater knowledge of the policy following exposure to the message labeled as strong (*M* = 5.40, *SD* = 1.45) rather than weak (*M* = 4.89, *SD* = 1.87). The Perceived Thought Type × Perceived Argument Quality interaction was also significant *F*(1, 283) = 5.35, *p* = 0.02, *n_p_^2^* = 0.02. Specifically, in the bolstering conditions, there was no difference in perceived knowledge when the message was labeled as weak (*M* = 5.14, *SD* = 1.80) or strong (*M* = 5.20, *SD* = 1.51), *F*(1, 283) = 0.04, *p* = 0.85, *d* = 0.03. However, in the counterarguing conditions, participants reported greater knowledge about the program when the message was labeled as strong (*M* = 5.61, *SD* = 1.36) rather than weak (*M* = 4.64, *SD* = 1.92), *F*(1, 283) = 12.14, *p* < 0.001, *d* = 0.58. Thus, similar to attitude certainty, perceived resistance strategy moderated the influence of argument quality on knowledge.^5^

#### Issue engagement

Similar to Experiment 1, participants reported a relatively high level of issue engagement and that was above the scale midpoint of 500 (*M* = 6.92, *SD* = 1.47, *t*(286) = 22.11, *p* < 0.001). A 2(Perceived Thought Type) × 2(Perceived Argument Quality) ANOVA on the issue engagement measure revealed no significant effects (*p*s > 0.09), suggesting that any differences in attitude certainty are not likely the result of differences in issue engagement of the message.

#### Intentions

A 2(Perceived Thought Type) × 2(Perceived Argument Quality) ANOVA on intentions revealed the predicted Perceived Thought Type × Perceived Argument Quality interaction *F*(1, 283) = 6.29, *p* = 0.01, *n_p_*^2^ = 0.02 (see Figure 2). Specifically, in the bolstering conditions, there was no difference in intentions to act on one’s attitude when the message was labeled as weak (*M* = 6.02, *SD* = 2.02) or strong (*M* = 5.67, *SD* = 2.20), *F*(1, 283) = 1.11, *p* = 0.29, *d* = 0.17. However, in the counterarguing conditions, participants reported greater intentions to act on their attitude when the message was labeled as strong (*M* = 6.61, *SD* = 1.83) rather than weak (*M* = 5.78, *SD* = 1.90), *F*(1, 283) = 6.27, *p* = 0.01, *d = 0*.45. Thus, similar to attitude certainty and perceived knowledge, perceived resistance strategy moderated the influence of argument quality on intentions to act.

### Mediation analyses

#### Attitude certainty as mediator on intentions

As noted earlier, the Perceived Thought Type × Perceived Argument Quality interaction affected attitude certainty and intentions. These results set up a test to examine whether, consistent with previous research, increases in attitude certainty from being exposed to a message labeled as strong perceived would lead to increases in intentions to act on one’s attitude (Tormala and Petty, 2002). Specifically, we expected that differences in attitude certainty would mediate the effect of argument quality on intentions. Importantly, we expect this possibility to occur in the counterarguing but not bolstering conditions. To test this, we conducted a moderated mediation analysis using bootstrapping procedures using the PROCESS macro for SPSS (Model 8; Hayes, 2013). The Perceived Thought Type × Perceived Argument Quality term was treated as the distal variable and attitude certainty term was treated as a potential mediator.

**Figure 3:**
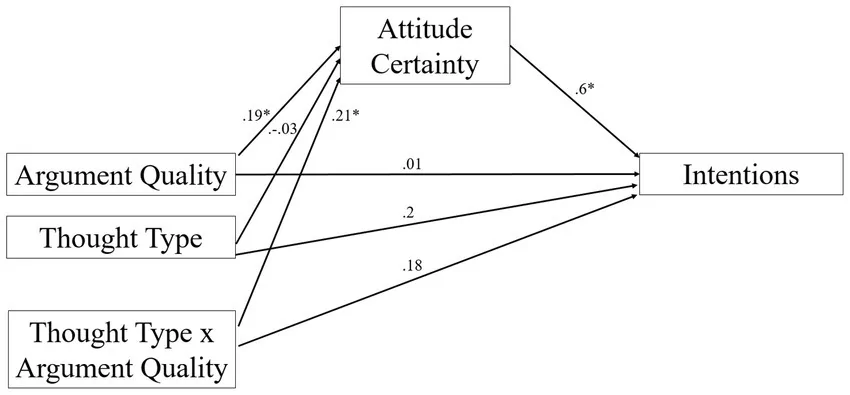
Moderated mediation of attitude certainty on perceived thought type × perceived argument quality effects on intentions. *p < 0.05.

Examination of the index of moderated mediation revealed that the higher order indirect effect of the attitude certainty term (*M* = 0.25, *SE* = 0.12) mediated the effect of the Perceived Thought Type × Perceived Argument Quality on the intention measure, 95% BS *CI*: [0.02, 0.51] (see Figure 3). The Perceived Thought Type × Perceived Argument Quality interaction on intentions was no longer significant, *b* = 0.17, *SE* = 0.11, *t*(282) = 1.71, *p* = 0.1, 95% CI: [−0.03, 0.38]. Thus, attitude certainty mediated the effect of the interaction between Perceived Thought Type and Perceived Argument Quality.^6^^,^^7^ We further decomposed the mediation by examining of the conditional indirect effect of perceived argument quality on intentions at each level of Perceived Thought Type. Results revealed that, when thoughts were perceived as counterarguments, the perceived argument quality effect on intentions was mediated by attitude certainty (*M* = 0.24, *SE* = 0.08 95% CI: [0.09, 0.41]). When thoughts were perceived as bolstering, however, no such mediation was evident (*M* = −0.02, *SE* = 0.09 95% CI: [−0.19, 0.17]).

## Discussion

To recap, Experiment 2 replicated the findings from Experiment 1: when thoughts were perceived as counterarguments, attitude certainty was greater in response to a strong than weak attack. However (and more novel), argument quality did not affect attitude certainty when thoughts were framed as bolstering thoughts. Moreover, participants’ attitudes were not affected by the feedback manipulation, suggesting that similar amounts of resistance (or lack of change) occurred across the conditions.

Experiment 2 also demonstrated that framing thoughts as counterarguments or bolstering thoughts has consequences for behavioral intentions. Specifically, when thoughts were perceived as counterarguments, attitudes became more predictive of behavioral intentions after participants had resisted a message believed to be strong, but not after they had resisted a message believed to be weak. Mediation analyses revealed that, consistent with previous research on certainty and intentions (Barden and Petty, 2008) certainty mediated the effect of argument quality on intentions, but only in the perceived counterargument conditions. Moreover, similarly high levels of issue engagement were found across the thought framing conditions, suggesting that participants amount of processing was not responsible for these attitude-intention effects.

## General discussion

Thoughts in response to a persuasive appeal can be integral to persuasion and resistance processes (Petty and Cacioppo, 1977; Chaiken et al., 1989). Despite the utility of the cognitive response approach in understanding a number of resistance strategies people use when exposed to counterattitudinal information, little research has examined the ways in which the different strategies are associated with persuasion and attitude strength. Previous work has demonstrated that, similar to persuasion, motivation and ability to resist an attack can create effortful and non-effortful forms of resistance that can affect the durability of the resistance (Wegener et al., 2004). The two experiments presented here advance this line of reasoning by demonstrating that two relatively thoughtful strategies, bolstering and counterarguing, can play an important role in attitude certainty following a persuasion attempt. Specifically, perceiving one’s thoughts as bolstering created post-message attitude certainty similar in magnitude to counterarguments, but were less sensitive to the quality of the arguments. Thoughts conceptualized as counterarguments elicited less attitude certainty when the counterattitudinal information was perceived as relatively weak. This decrease in certainty, in turn, led to a decreased willingness to act on the attitude. To our knowledge, this is the first evidence to demonstrate that attitude certainty can be differentially affected by the combination resistance strategies and argument quality.

The current experiments extend the work on resistance strategies (Jacks and Cameron, 2003; Briñol et al., 2004) from a metacognitive framework. That is, rather than comparing strategies on persuasion as the outcome of interest (as in McGuire, 1964), strategies were compared with an attitude strength feature (i.e., attitude certainty).^8^ This advance is part of a research program that highlights the importance of attitude features beyond valence and their application to the ways in which individuals actively try to maintain their attitudes (Wegener et al., 2019).

Second, the current experiments suggest that not all thought-based resistance strategies are equal with respect to their relation to an attack. When resisting an attack via counterarguing, the strength of the message seems to have a greater influence on attitude certainty than when resisting by bolstering.

Of course, we do not mean to suggest a bolstering strategy should be the preferred or even default defense in fending off an attack. The current experiments do not speak to the limiting conditions of bolstering, where such a strategy may be undermined. Future work should examine the boundary conditions for bolstering as a successful strategy. One possibility may be to consider situations where expressing one’s thoughts may be affected by other variables in a persuasion context that are not directly associated with the content of the attack. For example, sharing or advocating for a particular position to perceived experts may yield less certainty in bolstering thoughts than advocating to a less expert source, perhaps because they would be more open-minded to change their opinion (Ottati et al., 2015).

We believe that one unexpected result from Experiment 1 related to attitude bolstering merits mention. Specifically, participants in the bolstering feedback conditions had more favorable attitudes than in the counterargument feedback conditions. It could be that relative to perceiving thoughts as counterarguments, perceiving them as bolstering increased the perception that the thoughts were supportive of the initial position, thus making subsequent attitudes more extreme (i.e., more favorable). Such a finding is consistent with mere thought effects in self-persuasion (Clarkson et al., 2011). While this did finding did not replicate in Experiment 2, future work may help clarify whether perceiving thoughts as bolstering rather than as counterarguments may led to more extreme attitudes, which may translate into attitudes having a greater influence on behavior (Bechler et al., 2021).

One of the advantages of using the strategy manipulations in the current experiments is the experimental nature of the data to argue for causal effects of bolstering versus counterarguing effects on certainty. Specifically, we adapted a bogus feedback manipulation that targets participants’ perceptions of their thoughts on order to create a desired resistance strategy. While similar manipulations have been successful in affecting resistance success (Tormala and Petty, 2002) and attitude attributes such as value basis (Blankenship et al., 2022), we submit that it is unclear whether participants in the current experiments fully internalized the feedback. Therefore, in addition to devoting future research to exploring other means of manipulating resistance strategy (e.g., a bolstering mindset; Xu and Wyer, 2012), it would also be useful to implement the current manipulation in conjunction with a measure that assesses participants’ ability to internalize the feedback as a manipulation check.

Moreover, additional support for bolstering versus counterarguing effects on certainty and related attitude attributes could be gained by documenting relations between natural variations in the type of preferred resistance strategy on attitude certainty. That is, there are individual differences in the type of resistance strategy one prefers to use when defending an attitude (Jacks and Cameron, 2003; Briñol et al., 2004), and these differences are reflected in the type of thoughts generated in response to a counterattitudinal appeal (i.e., supporting vs. refuting; Briñol et al., 2004). As such, differences in thought content should yield similar results as the current experiments, such that “counterarguers” may be more sensitive to the quality of attacking information “bolsterers.” Future work should explore this possibility.

Future work could also examine various attitude-related features of resistance strategies. That is, in addition to attitude certainty, features such as issue importance and attitude-relevant knowledge may be associated with various strategies. For example, successfully generating counterarguments may require a relatively high amount of issue-relevant knowledge (perceived or actual), which may serve as ammunition in fending off an attack (Wood et al., 1995). Indeed, subjective perceptions of knowledge have been associated with the likelihood that one would use a counterarguing strategy to defend their position on an issue (Jacks and Cameron, 2003; Study 3). We included general measures of perceived knowledge in Experiment 2 to examine whether the strategies would influence knowledge. While participants did report greater knowledge when told that they counterargued a strong (rather than weak) attack, it may be fruitful to examine this relation further in future research.

Relatedly, it remains unclear which *type* of knowledge may be more closely associated with a counterarguing strategy. Because the majority of attitude-relevant knowledge is congenial to one’s opinions (Hart et al., 2009), much of the research on knowledge and attitude durability has focused on attitude-consistent knowledge (i.e., proattitudinal). As a result, very little research evidence has differentiated between knowledge that is supportive of versus in opposition to one’s position and their relation to resistance strategies. Burgeoning research differentiating between bolstering and counterarguing strategies such as the present experiments may help address this knowledge gap. That is, distinguishing between bolstering and counterarguing resistance strategies may have implications for the types of attitude-relevant knowledge one uses in defending their attitude. Specifically, knowledge of congenial information may be closely associated with bolstering and refutational strategies, in part because an accumulation of congenial knowledge would serve to support an attitude when attacked more than knowledge of uncongenial information. On the other hand, knowledge of uncongenial information (i.e., information that opposes one’s position) may be more closely associated with a refutational strategy, in part because knowledge of that information would likely be a source of effectively counterarguing information than congenial information. Using the previous hypothetical example, an individual who knows the flaws of efficiently extracting natural gas through fracking would find that information helpful in refuting an argument extolling the virtues of the efficiency of the fracking process.

To our minds, the current research addresses a relatively understudied component of resistance. Despite the potential importance of resistance strategies in understanding attitude strength (Jacks and Cameron, 2003; Albarracín and Mitchell, 2004; Fransen et al., 2015), there are surprisingly few investigations of how these strategies are employed in a persuasion context. Recent application of a metacognitive perspective in persuasion suggest that the appraisals derived from one’s lay theories of how information is processed and subsequent consequences have been fruitful in understanding how people view their information processing abilities (e.g., success, Tormala and Petty, 2004; bias correction, Wegener and Petty, 1997). The present research highlights that perceptions of the resistance strategies can influence features of attitude strength, depending on the characteristics of the attack. While the intent of the current experiments was not to examine all possible forms of strategies and attitude strength attributes, we hope that this research helps to ignite additional research on resistance strategies and their role in attitude strength and persuasion.

## Data availability statement

The datasets presented in this study can be found in online repositories. The names of the repository/repositories and accession number(s) can be found: https://osf.io/jc5n3/?view_only=a772c2458e884137805491450f115a28.

## Ethics statement

The studies involving human participants were reviewed and approved by the Iowa State University Institutional Review Board. The patients/participants provided their written informed consent to participate in this study.

## Author contributions

All authors listed have made a substantial, direct, and intellectual contribution to the work and approved it for publication.

## Conflict of interest

The authors declare that the research was conducted in the absence of any commercial or financial relationships that could be construed as a potential conflict of interest.

## Publisher’s note

All claims expressed in this article are solely those of the authors and do not necessarily represent those of their affiliated organizations, or those of the publisher, the editors and the reviewers. Any product that may be evaluated in this article, or claim that may be made by its manufacturer, is not guaranteed or endorsed by the publisher.

## Footnotes

^1^We report all measures, manipulations, and exclusions in the present text.

^2^A separate pilot study (*N* = 200) from the same student population found that 158 (79%) report opposing the university service proposal.

^3^In order to create a message that was moderate in strength (and not likely to create floor or ceiling effects), we adapted two arguments that were relatively specious (e.g., more money to beautify the campus) and two that were relatively cogent (e.g., ensure that a college education would remain affordable), based on previous research (Baker and Petty, 1994).

^4^Previous research has demonstrated that the facets of attitude certainty have different antecedents and consequences (Petrocelli et al., 2007). For exploratory purposes, we conducted analyses on the separate certainty subscales. A Thought × Argument quality ANOVA on the attitude clarity measure (with attitude correctness as a covariate) revealed a significant interaction *F*(1, 283) = 6.20, *p* = 0.01, *n_p_*^2^ = 0.02, with attitude clarity higher in the strong than weak argument conditions in the counterarguing but not bolstering conditions. A similar analysis on the attitude correctness measure (with attitude clarity as a covariate) yielded no significant effects (*p*s > 0.36). While tentative, it may be that the attitude clarity aspect of attitude certainty is driving the difference rather than correctness.

^5^Because of the relatively low correlation between the two knowledge items, we conducted the same factorial ANOVA on each item separately. Results indicated that the patterns of results were largely the same as the combined measure (interaction *p*_s_  = 0.05 and 0.068 for the attitude knowledge and issue knowledge items, respectively).

^6^We also conducted an exploratory analysis to examine whether perceived knowledge was a mediator on intentions. We conducted a similar conditional moderation analyses described in the text. The higher order indirect effect of the knowledge term (*M* = 0.21, *SE* = 0.1) mediated the effect of the Thought Type × Argument Quality on the intention measure 95% BS CI: [0.03, 0.42]. The Thought Type × Argument Quality interaction on intentions was no longer significant, *b* = 0.19, *SE* = 0.11, *t*(281) = 1.78, *p* = 0.08, 95% CI: [−0.02, 0.41].

^7^We also examined whether perceived knowledge was a mediator on certainty using a similar conditional moderation analyses described in the text with certainty as the outcome. The higher order indirect effect of the knowledge term (*M* = 0.26, *SE* = 0.12) mediated the effect of the Thought Type × Argument Quality on certainty 95% BS CI: [0.04, 0.51]. The Thought Type × Argument Quality interaction on certainty was no longer significant, *b* = 0.08, *SE* = 0.08, *t*(281) = 0.99, *p* = 0.32, 95% CI: [−0.08, 0.23]. While tentative, these results suggest that participants’ inferences about their knowledge about the topic influenced perceptions of attitude certainty when their thoughts were perceived as counterarguments (Tormala and Petty, 2002, p. 1300).

^8^While previous research has found that different strategies vary in post-attack certainty (e.g., Tormala et al., 2007), the strategies likely differed in other ways (e.g., amount of effort, source focus vs. message focus, etc.).
